# Sex-Specific Effects of Acute Ethanol Exposure on Locomotory Activity and Exploratory Behavior in Adult Zebrafish (*Danio rerio*)

**DOI:** 10.3389/fphar.2022.853936

**Published:** 2022-06-02

**Authors:** Laura E. Vossen, Ronja Brunberg, Pontus Rådén, Svante Winberg, Erika Roman

**Affiliations:** ^1^ Division of Anatomy and Physiology, Department of Anatomy, Physiology and Biochemistry, Swedish University of Agricultural Sciences, Uppsala, Sweden; ^2^ Neuropharmacology, Addiction and Behavior, Department of Pharmaceutical Biosciences, Uppsala University, Uppsala, Sweden; ^3^ Behavioral Neuroendocrinology, Department of Neuroscience, Uppsala University, Uppsala, Sweden; ^4^ Behavioral Neuroendocrinology, Department of Medical Cell Biology, Uppsala University, Uppsala, Sweden

**Keywords:** alcohol, swimming kinematics, exploration, anxiety-like behavior, sex differences, multivariate concentric square field (MCSF), risk taking, shelter seeking behaviour

## Abstract

The zebrafish (*Danio rerio*) is an established model organism in pharmacology and biomedicine, including in research on alcohol use disorders and alcohol-related disease. In the past 2 decades, zebrafish has been used to study the complex effects of ethanol on the vertebrate brain and behavior in both acute, chronic and developmental exposure paradigms. Sex differences in the neurobehavioral response to ethanol are well documented for humans and rodents, yet no consensus has been reached for zebrafish. Here, we show for the first time that male zebrafish of the AB strain display more severe behavioral impairments than females for equal exposure concentrations. Adult zebrafish were immersed in 0, 1 or 2% (v/v) ethanol for 30 min, after which behavior was individually assessed in the zebrafish Multivariate Concentric Square Field™ (zMCSF) arena. Males exposed to 2% ethanol showed clear signs of sedation, including reduced activity, increased shelter seeking and reduced exploration of shallow zones. The 1% male group displayed effects in the same direction but of smaller magnitude; this group also explored the shallow areas less, but did not show a general reduction in activity nor an increase in shelter seeking. By contrast, 1 and 2% exposed females showed no alterations in explorative behavior. Females exposed to 2% ethanol did not display a general reduction in activity, rather activity gradually increased from hypoactivity to hyperactivity over the course of the test. This mixed stimulatory/depressant effect was only quantifiable when locomotory variables were analyzed over time and was not apparent from averages of the whole 30-min test, which may explain why previous studies failed to detect sex-specific effects on locomotion. Our results emphasize the importance of explicitly including sex and time as factors in pharmacological studies of zebrafish behavior. We hypothesize that the lower sensitivity of female zebrafish to ethanol may be explained by their greater body weight and associated larger distribution volume for ethanol, which may render lower brain ethanol concentrations in females.

## 1 Introduction

Alcohol use disorders (AUDs) and alcohol-related disease have a severe negative impact on individual health and social functioning, and on society. It is estimated that worldwide, 3 million deaths every year occur as a result of the harmful use of alcohol (Poznyak and Rekve, 2018). Alcohol (ethanol, EtOH) has both acute and chronic effects on the brain and on behavior, which to a large extent has been characterized using rodents as model organisms (Bell et al., 2017). Ethanol has a highly complex mechanism of action (Koob et al., 1998; Abrahao et al., 2017), therefore much remains to be investigated. In recent years, the zebrafish has gained popularity as a model organism in biomedical research owing to its short developmental time, ease of maintenance, high egg production, external fertilization and possibilities for gene editing tools (Liu et al., 2019). Given the increasing use of zebrafish it is important to translate and confirm results from rodents to zebrafish.

Ethanol can be administered to zebrafish via the holding water for studies of both acute (Gerlai et al., 2000) and chronic effects (Gerlai et al., 2006; Gerlai et al., 2009) as well as developmental ethanol exposure (Carvan et al., 2004; Lockwood et al., 2004). The most commonly used doses are immersion treatments with 0.25, 0.5 and 1.0% (v/v) ethanol for one hour, which result in blood and brain ethanol concentrations that are comparable to human drinkers after mild to moderate acute ethanol consumption (Dlugos and Rabin, 2003; Echevarria et al., 2011; Rosemberg et al., 2012). In agreement with mammals (Pohorecky, 1977; Calabrese and Baldwin, 2003), acute ethanol exposure alters zebrafish locomotory behavior in a biphasic dose-dependent manner; low concentrations (0.25 and 0.5% for 60 min) increase locomotory activity while higher concentrations (1% for 60 min) result in reduced activity (Gerlai et al., 2000; Gerlai, 2003), suggesting a sedative effect (but see (Gerlai et al., 2006; Mathur and Guo, 2011)). Moreover, increases in risk-taking behavior have been reported for the 0.5 and 1% dose (60 min immersion duration), including a reduced avoidance reaction (Gerlai et al., 2006; Gerlai et al., 2009), reduced diving response (Gerlai et al., 2008; Mathur and Guo, 2011), reduced scototaxis (Gebauer et al., 2011; Mathur and Guo, 2011) and reduced shoaling behavior (Gerlai et al., 2009). Finally, considerable differences in the behavioral responses to ethanol have been found between zebrafish strains (Gerlai et al., 2008; Gerlai et al., 2009; Pannia et al., 2014), emphasizing the importance of genetic differences.

Although zebrafish males and females can be difficult to distinguish from one another morphologically, it is well recognized that the sexes display behavioral differences in locomotory activity, exploration, boldness and aggression (Dahlbom et al., 2011; Dahlbom et al., 2012; Mustafa et al., 2019; Genario et al., 2020; Dos Santos et al., 2021; Souza et al., 2021). The sexes also differ markedly in responses to various toxicants and pollutants (reviewed in (Genario et al., 2020)), although for the case of ethanol no consensus has been reached. While most acute exposure studies used a population containing both sexes, and in many cases even reported an equal sex ratio, results from both sexes were often pooled in the statistical analysis (but see (Dlugos et al., 2011; Clayman et al., 2017; Goodman and Wong, 2020; Souza et al., 2021)). One exception is a study by Dlugos *et al.* (2011) who reported a greater increase in nearest neighbor distance in groups of wild-type females compared to males after exposure to 0.5% ethanol for 2 h, while males showed a greater increase at the 1% (v/v) dose (Dlugos et al., 2011). In addition, a recent study by Souza et al. (2021) found that the behavior of short-fin zebrafish females towards a novel object was affected by low concentrations (0.25 and 0.5% (v/v) for one hour) where male behavior was unaffected. We asked the question, to what extend the sex differences reported extrapolate to other behavioral contexts and zebrafish strains, in particular to the widely used AB strain. We wanted to include several proxies for ethanol-induced effects on activity, exploration, shelter seeking and risk-taking behavior while avoiding carry-over and test order effects between different behavioral tests. We therefore chose to use the recently developed zebrafish version of a multivariate test arena used in over 40 rodent studies, the Multivariate Concentric Square Field™(MCSF) (Bikovski et al., 2020; Vossen et al., 2022).

The zebrafish MCSF (zMCSF) arena consists of a central square area surrounded by semi-sheltered corridors, a dark corner roof (DCR), and an inclined ramp (Vossen et al., 2022). These areas differ in illumination and elevation, are sheltered or exposed to different degrees, and the arena cannot be overseen from any of these areas. This design offers the animal a free choice between several alternative locations of different quality in terms of risk and safety, while also providing an incentive for exploration. This generates a behavioral profile of an individual within a single behavioral test. In a previous study, we repeatedly tested male and female AB and wild-caught zebrafish in this arena, and cross-validated the results with the novel tank diving test (Vossen et al., 2022). This revealed no major sex differences in exploratory behavior, except for a higher locomotory activity in AB males compared to females. However, we detected large differences between strains. AB zebrafish avoided the risky area (inclined ramp), and often left one or more zones unexplored. Wild zebrafish swam faster than AB and spent more time on the ramp, but avoided the center of the arena. The zMCSF was largely resilient to repeated testing. These results led us to conclude that the zMCSF can distinguish between different magnitudes and types of risk taking and shelter seeking, which may also render a more fine-grained analysis of the changes in behaviors induced by acute ethanol exposure (Vossen et al., 2022).

The aim of the current study was to investigate whether acute ethanol exposure differentially affected behavioral profiles of male and female AB zebrafish in the zMCSF. In previous studies, the effect of 1% (v/v) acute ethanol exposure on locomotory activity was inconsistent even within the AB strain (Gerlai et al., 2008; Mathur and Guo, 2011; Tran and Gerlai, 2013) and AB appeared to be more tolerant than other strains (Gerlai et al., 2008). We therefore chose to include one widely used dose (1% v/v) and one higher dose (2% v/v) as suggested by Gerlai and coworkers (Gerlai et al., 2008). Regarding the choice of immersion duration, two zebrafish studies have reported that brain and blood ethanol content reached a steady-state concentration within 15–20 min (Dlugos and Rabin, 2003; Echevarria et al., 2011) while another study saw a small but significant increase from 15 to 30 min (Rosemberg et al., 2012). Hence, we chose an immersion duration of 30 min.

## 2 Materials and Methods

Experiments took place at the Department of Neuroscience, located at the Biomedical Center of Uppsala University, Sweden in October and November 2017. Ethical approval for the use of animals was given by the Uppsala Regional Animal Ethical Committee (permit C55/13), following the guidelines of the Swedish Legislation on Animal Experimentation (Animal Welfare Act SFS 1998:56) and the European Union Directive on the Protection of Animals Used for Scientific Purposes (Directive 2010/63/EU).

### 2.1 Animals and Housing

A total of 51 adult zebrafish (23 females and 28 males, 19 months old) of the AB strain were used in this study. Animals were obtained from SciLifeLab (Evolutionary Biology Center, Uppsala University), a local zebrafish facility that regularly obtains AB strain zebrafish from the Zebrafish International Resource Center (ZIRC at the University of Oregon Eugene). Animals were kept in mixed-sex groups in a stand-alone system (Aquaneering, San Diego, 117 United States) in 2.8L tanks supplied with recirculating copper-free Uppsala municipal tap water (10% daily exchange). Temperature was maintained at 27 ± 1.5 °C and the photoperiod was 14L:10D (lights on at 07:00 a.m.). Animals were fed twice a day with flakes (tropical energy food, Aquatic 120 Nature, Roeslare, Belgium) and *Artemia* brine shrimp (Argentemia Platinum Grade 0, Argent 121 Aquaculture, Redmond, United States). All animals were naïve to behavioral testing.

### 2.2 Ethanol Exposure

One week before the start of behavioral testing, subjects were randomly placed into one of three 2.8L rack system tanks (Aquaneering, San Diego, United States); tank 1 (9 females, 9 males), tank 2 (10 females, 10 males) or tank 3 (9 males, 9 females). The sex of each individual was determined by visual examination (in brief, extrusion of belly, ovipositor and color of the anal fin (Gupta and Mullins, 2010)). The housing tanks were kept in the rack system for 7 days prior to behavioral testing, to ensure a stable dominance hierarchy within each dose group. Immediately prior to behavioral testing, each fish was individually immersed for 30 min into a 1.75L trapezoidal tank containing 1.0L rack system water mixed with 0, 1 or 2% (v/v) ethanol (≥99.5%, VWR, Sweden). Exposure started no earlier than 30 min after morning feeding. Immediately following ethanol (or control) treatment, an individual zebrafish was transferred to the zMCSF arena using a small net, and its behavior was video recorded for 30 min.

### 2.3 The Zebrafish Multivariate Concentric Square Field™ (zMCSF)

**FIGURE 1:**
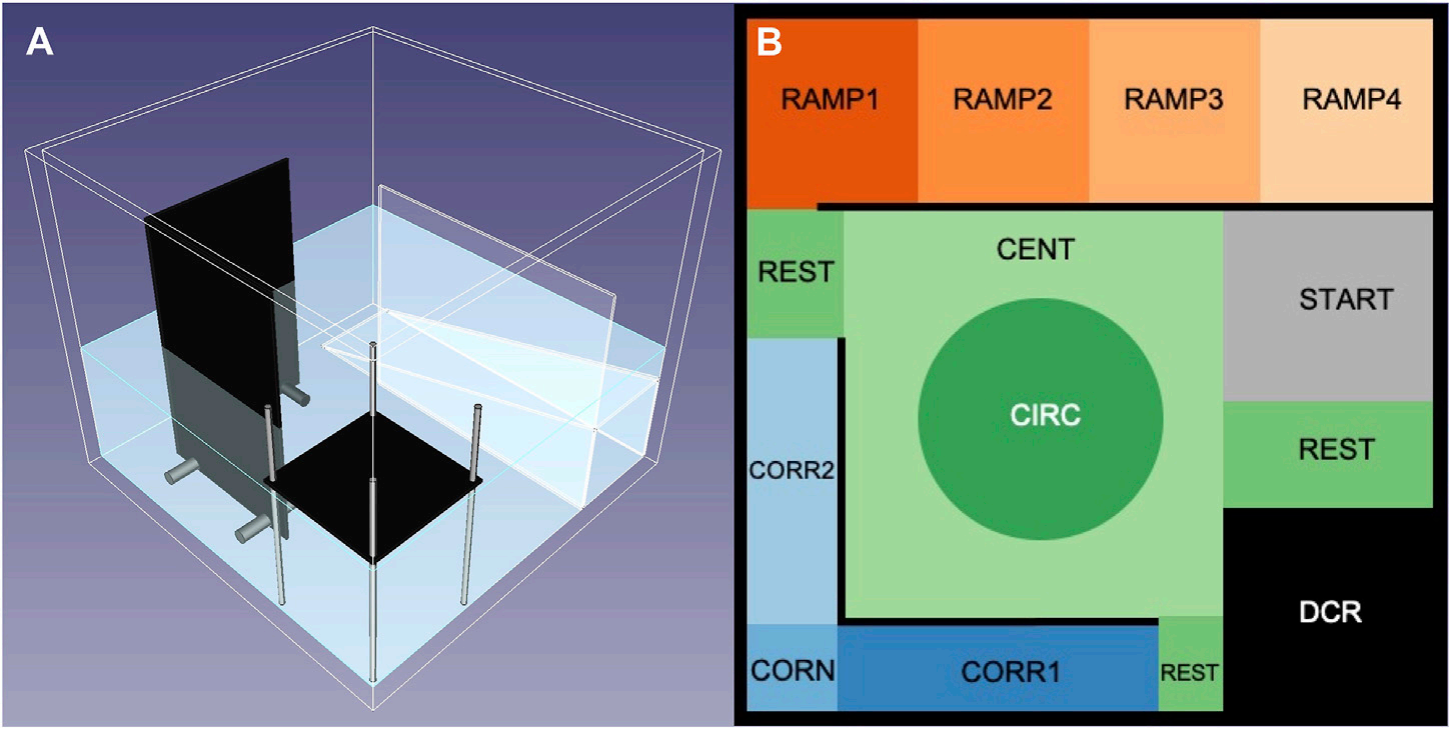
The zMCSF testing arena, which contains a dark corner roof (DCR), two walls building a corridor and corner (CORR1, CORN, CORR2), and an inclined ramp creating decreasing water depth (RAMP1-4), all of which surround a central open area (CENT and CIRC). For exact measurements, see blueprints provided in (Vossen et al., 2022). **(A)** 3D model of the zMCSF arena. **(B)** Virtual division of zones in the arena, as seen by the ceiling mounted camera and used for video tracking with Ethovision XT15 (Noldus, Wageningen, the Netherlands). Images reprinted from (Vossen et al., 2022). Abbreviations: CENT, center; CIRC, central circle; CORN, corner; CORR, corridor; DCR, dark corner roof; REST, the part of the arena not designated to any other zone.

The multivariate concentric square field is a behavioral test arena that was originally developed for rodents (Bikovski et al., 2020; Meyerson et al., 2006). We recently translated the MCSF test to zebrafish and quantified strain and sex differences as well as the effect of repeated testing (Vossen et al., 2022). All behavioral tests took place in a separate room located inside the aquarium room. The experimenter was not present or visible during video recordings. The zMCSF is a square tank (30 × 30 × 25.8 cm) containing three objects; a roof, a corridor and a ramp, which are placed around the walls thereby surrounding a central open area (Figure 1). The arena is filled with 8L pre-heated copper-free Uppsala municipal tap water (23 ± 2°C) creating a water depth of 10 cm. The water inside the arena is exchanged fully between trials. An infrared backlight (Noldus, Wageningen, Netherlands) is placed under the zMCSF arena and an infrared camera (JVC SuperLoLux, Yokohama, Japan) on the ceiling records the movement of the fish through the arena. Two photographic lights (Walimex daylight 1,000, the Hague, Netherlands) provide ambient lighting of 0.46 Lux (Lux meter, Fisher Scientific LTD., Uppsala, Sweden). The arena is divided (virtually) into 13 zones (Figure 1): the area where the animal is released into the arena (START), a dark corner with a roof (DCR), a semi-sheltered area consisting of two corridors (CORR1 and CORR2) and a corner (CORN), an inclined ramp leading from high to low water depth (RAMP1-4), a central square consisting of an central circle (CIRC) and the remnant of the central square (CENT) and finally the remaining floor area that does not belong to any of the other zones (REST). From previous work on the zMCSF we derived that zebrafish seek shelter in the DCR (and to some extend CORR1, CORN and CORR2), while the RAMP zones (especially RAMP4) comprise high risk zones for AB zebrafish (Vossen et al., 2022).

### 2.4 Video Tracking and Data Extraction

Videos were recorded and video tracked with Ethovision XT12 software (Noldus, Wageningen, the Netherlands). Behavioral recording started 2 s after the animal was detected in the arena and ended 30 min later. All trials were manually assessed for possible tracking errors. We extracted six variables from the tracks using Ethovision XT15 (Noldus, Wageningen, Netherlands). For the whole arena we extracted duration in arena, total distance moved (cm) and average velocity (cm s^−1^). For each zone, we extracted the cumulative duration (s) in zone, frequency of zone visits and latency (s) until first entry into zone. For the REST zone only the duration (s) in this zone could be extracted. From these variables, we computed an additional five ethologically relevant variables (Vossen et al., 2022). Total activity was defined as the sum of all zone frequencies (zone entries). Duration per visit (s) was computed as the total duration in zone divided by the frequency of visits to that zone. Frequency (%) was calculated as the frequency of visits to that zone divided by total activity. Using the latency (s) variable, we derived the number of zones entered by the fish, and if the fish had explored all zones (yes or no). Finally, these same variables were extracted from Ethovision over time, with one minute per time bin, which was labeled the ‘minute bins dataset’.

### 2.5 Statistical Analyses

All statistical analyses were carried out in R statistical computing software version 4.0.2 (R_Core_Team, 2020) with added packages “lme4” (Bates et al., 2015), “emmeans” (Lenth, 2020) “bestNormalize” (Peterson and Cavanaugh, 2019; Peterson, 2021) and “ggplot2” (Wickham, 2016). We first explored the data by conducting a principal component analysis (PCA) on each dataset, using the “prcomp” function with scaling and centering of variables.

#### 2.5.1 Statistical Analysis of Locomotory Activity

To evaluate the effect of ethanol dose (hereafter Dose) and the sex of the individual (hereafter Sex) on total distance moved (cm) and mean velocity (cm s^−1^) over the whole trial, we computed two two-way ANOVAs with main effects of Dose and Sex plus the interaction effect. Total activity was modelled with a generalized linear model (GLM) with a negative binomial error distribution, using the same explanatory variables. Post-hoc pairwise comparisons with Bonferroni correction for multiple testing were computed using the “emmeans” function.

Since these three activity variables followed a linear pattern over time, this allowed for regression analyses on the minute bin dataset using mixed-effect modeling which allows for inclusion of repeated measurements on the same individuals. Distance moved (cm) per minute and velocity (cm s^−1^) per minute were analyzed with two linear mixed-effect models (LMMs) with fixed effects of Dose and Sex and Minute and a random intercept of Individual. The “emtrends” function was used to compare the slopes of the different Dose/Sex groups. Total activity per minute was modelled using a generalized linear mixed-effects model (function “glmer.nb”) incorporating the same explanatory variables as for distance moved (cm) and velocity (cm s^−1^) per minute.

#### 2.5.2 Statistical Analysis of Exploratory Behavior, Risk Taking and Shelter Seeking

To evaluate exploratory behavior, we constructed a Poisson GLM with the number of zones explored as a response variable and Dose, Sex and their interaction as explanatory variables. In addition, a binomial GLM with the same explanatory variables was performed on the binary variable indicating whether all zones had been visited.

Risk taking and shelter seeking were evaluated using zone-specific variables total duration (s), average duration per visit (s) and latency (s). A linear mixed-effects model (LMM) was constructed with fixed effects of Zone, Dose and Sex and their interactions and a random intercept of Individual. We applied a log transformation on duration and duration per visit (log (y+1)). Latency was transformed using an ordered quantile normalization (“orderNorm” function (Peterson and Cavanaugh, 2019)), a rank-based procedure, suggested by the “bestNormalize” function (Peterson, 2021). For count variables frequency and frequency (%) we computed a negative binomial generalized linear mixed-effects model (GLMM), with the same fixed and random effects, after a Poisson GLMM proved to suffer from overdispersion. For all models, we computed post-hoc comparisons with Bonferroni correction and we tested only for effects within each Zone between all Dose/Sex groups.

The response variables did not show a linear pattern over time (i.e. per minute), therefore the minute bins dataset could not be analyzed using (G)LMMs. However, we provide the reader with detailed graphs of duration (s) and frequency in zone per Dose/Sex group for visual analysis.

## 3 Results

### 3.1 Principal Component Analysis (PCA)

Individual principal component scores largely overlapped between groups (Supplementary Figure S1A). Only the 2% male group stood out, having a relatively low score on PC1 and PC2 score, indicative of a longer duration in DCR and low activity. The loading plot showed a clear separation of the variables from each zone (Supplementary Figure S1B).

### 3.2 Locomotory Activity

**FIGURE 2:**
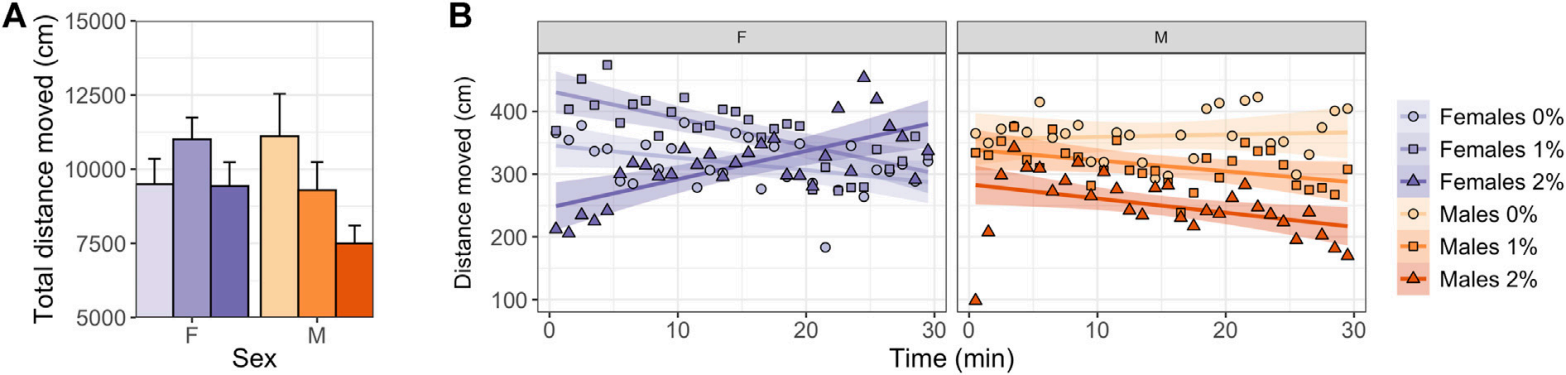
Locomotory activity in the zMCSF of male and female AB zebrafish acutely exposed to 0, 1 or 2% (v/v) ethanol for 30 min. **(A)** Total distance moved over the 30-min trial (in cm; mean ± SEM per group) and **(B)** distance moved per minute (in cm; mean per group) by female (F; purple colors) and male (M; orange colors) zebrafish exposed to 0% (controls; light shades), 1% ethanol (medium dark shades) or 2% ethanol (dark shades). Lines indicate the linear regression line and confidence intervals (shaded) per Dose/Sex group.

Males exposed to 2% ethanol showed a reduction in total distance moved (ANOVA, t_2,45_ = 2.755, *p* = 0.025; Figure 2A, Supplementary Table S1, S2) and mean velocity compared to control males (ANOVA, t_2,45_ = 2.764, *p* = 0.025; Supplementary Table S1, S2). No dose differences were observed in females over the entire 30-min test (Figure 2A, Supplementary Table S1, S2).

Regression analyses over time revealed sex-specific effects of Dose on activity patterns (Tables 1, 2, 3 and Figure 2B). Both 1% and 2% males showed a more negative slope in distance moved over time compared to control males (LMM contrasts; males 0 vs 1%: t_2,1473_ = 3.256, *p* = 0.014; t_2,1473_ = 3.344, *p* = 0.014; Table 3 and Figure 2B). While control and 1% females showed a decrease in distance moved over time, 2% females had a positive slope in distance moved over time (LMM contrast; females 0 vs 2%: t_2,1473_ = -4.960, *p* < 0.001; 1 vs 2%: t_2,1473_ = -6.781, *p* < 0.001; Table 3 and Figure 2B). Finally, the slope of males and females differed significantly for the control dose (LMM contrast; t_2,45_ = -3.070, *p* = 0.013) and 2% dose (LMM contrast, t_2,45_ = 6.780, *p* < 0.001), but not for the 1% dose (LMM contrast; t_2,45_ = -2.200, *p* = 0.068; Table 3 and Figure 2B). The patterns in velocity and total activity were highly similar to distance moved (Supplementary Table S2).

**TABLE 1:** Results of the linear mixed-effects model of Distance moved (cm) per minute, with fixed effects of Minute, Dose and Sex and all interactions, and a random intercept of individual.

Response	Explanatory	Test statistic	df	p-value	
Distance moved (cm)	Dose	F = 2.483	2, 61	0.087	-
	Sex	F = 0.714	1, 61	0.398	-
	Minute	F = 8.180	1, 1,473	0.004	**
	Dose × Sex	F = 2.151	2, 61	0.116	-
	Dose × Min	F = 10.648	1, 61	<0.001	***
	Sex × Min	F = 0.193	1, 1,473	0.660	-
	Dose × Sex × Min	F = 18.788	2, 1,473	<0.001	***

**TABLE 2:** Post-hoc pair-wise comparisons between Dose/Sex groups in average Distance moved (cm) per minute, averaged over all Minutes.

	Contrast	t ratio	df	p-value	
Females	0–1%	−1.085	2, 45	0.851	-
	0–2%	0.041	2, 45	1.000	-
	1–2%	1.089	2, 45	0.846	-
Males	0–1%	1.422	2, 45	0.486	-
	0–2%	2.755	2, 45	0.025	*
	1–2%	1.405	2, 45	0.501	-
Sex difference	0%	−1.198	2, 45	0.237	-
	1%	1.294	2, 45	0.202	-
	2%	1.380	2, 45	0.174	-

**TABLE 3:** Post-hoc pair-wise comparisons of the slope in Distance moved over time, per Sex/Dose group.

	Contrast	t ratio	df	p-value	
Females	0–1%	1.885	2, 1,473	0.144	-
	0–2%	−4.960	2, 1,473	<0.001	***
	1–2%	−6.781	2, 1,473	<0.001	***
Males	0–1%	2.798	2, 1,473	0.014	*
	0–2%	2.802	2, 1,473	0.014	*
	1–2%	0.076	2, 1,473	0.997	-
Sex difference	0%	−3.070	2, 45	0.013	*
	1%	−2.200	2, 45	0.068	-
	2%	6.780	2, 45	<0.001	***

#### 3.2.1 Exploratory Behavior, Risk Taking and Shelter Seeking

**FIGURE 3:**
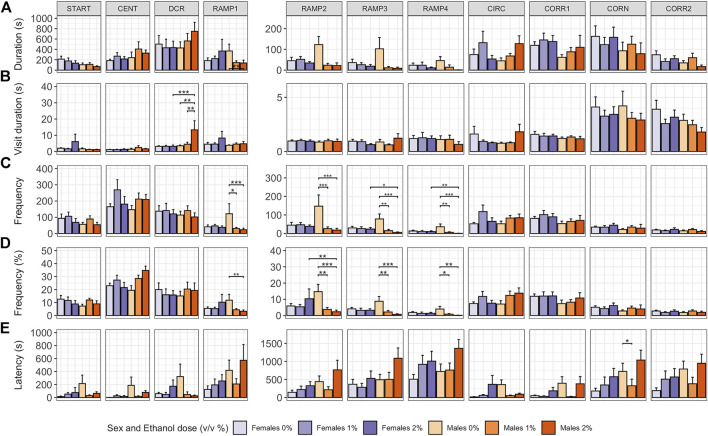
Exploration of the zMCSF arena by male and female AB zebrafish acutely exposed to 0, 1 or 2% (v/v) ethanol for 30 min. Within each graph, the data is presented per Dose/Sex group. The different zones are presented in different columns. Rows contain different response variables: **(A)** Duration in zone (s), **(B)** Duration per visit in zone (s), **(C)** Frequency of zone entries, **(D)** Frequency of zone entries (as percentage of the total number of zone entries) and **(E)** Latency (s) until first entry into a zone. Colors indicate Dose/Sex group as follows: females (purple colors) and males (orange colors) zebrafish exposed to 0% (controls; light shades), 1% ethanol (medium dark shades) or 2% ethanol (dark shades). Bars represent mean ± SEM over the 30-min trial. Stars indicate significant differences (*p < 0.05, **p < 0.01, ***p < 0.001).

In males, ethanol dose influenced whether or not a fish explored all zones of the zMCSF (Poisson GLM, χ^2^_2,48_ = 7.492, *p* = 0.024; Supplementary Table S2). While for 1% males 9 out of 10 animals explored all zones, in 2% males, only 3 out of 9 animals explored all zones (Supplementary Table S1). Both ethanol dose and the sex of the fish influenced how long and how often animals visited the zones of the zMCSF, as indicated by significant three-way interactions between Zone, Dose and Sex for variables duration in zone, frequency in zone and percentage frequency in zone (Supplementary Table S1, S2). Compared to control males, 2% males spent shorter durations in RAMP1-4 (LMM contrasts; t_22,309_ = 3.215, *p* = 0.022; t_22,309_ = 3.612, *p* = 0.005; t_22,309_ = 3.599, *p* = 0.006; t_22,309_ = 3.186, *p* = 0.024; Figure 3A and Supplementary Table S1) and a longer duration per visit in the DCR (LMM contrast, t_22,392_ = -3.974, *p* = 0.001; Figure 3B and Supplementary Table S1). Graphical analysis of duration and frequency in zone over time revealed that the long visits to the DCR by the 2% males were particularly pronounced in the last 10 minutes of the test, a time at which control males explored RAMP1-4 (Supplementary Figure S2). While the 1% males also entered RAMP1-4 less often (LMM contrasts; t_22,309_ = 3.288, *p* = 0.015; t_22,309_ = 4.195, *p* < 0.001; t_22,309_ = 3.872, *p* = 0.002; t_22,309_ = 3.968, *p* = 0.001; Figures 3C,D and Supplementary Table S1), they did not pay long visits to the DCR (LMM contrast, t_22,392_ = -0.345, *p* = 1.000; Figure 3B and Supplementary Table S1).

In contrast to males, in females pairwise comparisons between control, 1% and 2% doses did not reveal any significant differences in zone-related variables (Figure 3 and Supplementary Table S1). Control females furthermore did not differ from control males for any zone or variable (Figure 3 and Supplementary Table S1). Although all female groups paid shorter visits to the DCR than all male groups (LMM contrast, t_10,383_ = -3.372, *p* = 0.001), this effect was driven by the long visits to the DCR by the 2% males.

## 4 Discussion

In the present study, we exposed male and female zebrafish to control, 1% or 2% ethanol (v/v) for 30 min, after which we behaviorally phenotyped animals in the zMCSF test that we recently described (Vossen et al., 2022). Ethanol treatment differentially affected the behavior of female and male AB zebrafish. The strongest effects of the acute ethanol exposure were seen in the 2% male group, which showed a significant reduction in locomotory activity throughout the entire test, reduced risk taking, as indicated by spending less time on the inclined ramp and increased shelter seeking by longer duration per visit to the DCR. The 1% male group displayed effects in the same direction but of smaller magnitude; this group also explored the inclined ramp less, while no general reduction in activity nor an increased duration per visit to the DCR could be detected. Females exposed to 2% ethanol showed signs of lower activity during the first 10 minutes of the test, but gradually increased their activity over the test and became more active than both control and 1% females in the last 10 minutes. Explorative behavior was unaffected in 2% females. The 1% exposed females showed no alterations in any of the measured behaviors.

### 4.1 Sex Differences in the Response to Acute Ethanol Exposure

The observed effects of both the 1% and 2% dose on males may be best interpreted as sedative effects, which likely arise from the well-established motor suppressing and intoxicating effect of high ethanol doses (Koob et al., 1998). Although a reduced time on the ramp and longer visits to the DCR are usually interpreted as lower risk-taking and increased shelter seeking, respectively (Meyerson et al., 2013; Vossen et al., 2022), an alternative explanation is that the sedated males moved to the DCR to rest, thereby inevitably reducing the time spent on ramp. Indeed, we previously found that the majority of AB zebrafish choose the DCR as their “home base” (Vossen et al., 2022), i.e. a location in which the animal spends a disproportional amount of time and from which it makes round trips in different directions, which is often the preferred area for rest or sleep (Eilam and Golani, 1989; Stewart et al., 2010).

The mixed stimulatory/depressant effects on locomotory activity found in 2% females, with initial low activity followed by hyperactivity, show a striking resemblance to that of female mice exposed to an intermediate dose (Matchett and Erickson, 1977). While a low dose (0.5 or 1.0 g/kg i. p.) had mild stimulatory effects and a high dose (4.0 g/kg i. p.) produced a general reduction in activity, an intermediate dose (2.0 g/kg i. p.) produced first sedative followed by stimulatory effects on spontaneous locomotory behavior (Matchett and Erickson, 1977). It is possible that zebrafish females quickly eliminated the high ethanol dose from the blood (by ventilation or enzymatic oxidation, or both), whereby the lower concentration at the end of the test produced the stimulatory effect on locomotion. To test this hypothesis, more data on the rate of ethanol elimination in male and female zebrafish are needed. Hence, it seems that 2% ethanol was an intermediate dose for female zebrafish while it constituted a high (sedative) dose for males. In other words, the dose-response curve of female zebrafish was shifted towards the right compared to males, making females the less affected sex in this strain and possibly, in this species. To further map the dose-response curve of both sexes, we suggest to behaviorally test female AB zebrafish at doses above 2% to capture at what dose sedative effects occur. In males, concentrations below 1% may convey at what concentration stimulatory and mixed stimulatory/depressant effects occur. It may also be necessary to increase the test duration to be able to estimate recovery periods for all doses.

The sex differences observed in AB zebrafish may be partially explained by the larger standardized weight of females compared to males (Fulton’s condition factor (Fulton, 1902)). In a recent study using AB zebrafish from the same supplier (Vossen et al., 2020), we found females to have a 27% larger standardized weight (L.E. Vossen, unpublished data). This might well translate into a larger aqueous volume which renders a larger distribution volume for ethanol in females, reducing blood alcohol concentrations (Smith et al., 2013). Alternative explanations, such as a sex differences in absorption (Klockhoff et al., 2002), metabolizing enzymes present in the liver and stomach (Frezza et al., 1990; Baraona et al., 2001), an interaction effect with sex hormones (Dettling et al., 2008) or actual sex differences in the sensitivity of the brain at equal blood alcohol concentrations (Miller et al., 2009), as evident from human studies, should certainly not be excluded. An important recent discovery concerns the role of a brain metabolic pathway of ethanol in producing behavioral effects typical of intoxication (Jin et al., 2021).

### 4.2 Lack of Sex Differences in Control Animals

We observed few sex differences in unexposed AB strain females and males in the zMCSF, in line with our earlier study (Vossen et al., 2022). At baseline, control males increased their activity over time while control females showed a slight decrease, but we found no sex differences in activity or exploration over the whole trial. In conventional behavioral tests, female zebrafish often display a more “shy” or “reactive” stress coping style than males. Although there are considerable differences between strains and tests, females tend to show increased levels of thigmotaxis and shelter seeking (Dahlbom et al., 2011; Mustafa et al., 2019), a stronger diving response (Mustafa et al., 2019), lower activity in a novel environment (Mustafa et al., 2019) and more hesitation towards a novel object (Souza et al., 2021) compared to males. The absence of a sex difference in control animals in the zMCSF may be explained by the design of this arena (Vossen et al., 2022). Since males often display a higher activity in conventional tests, this may “drive” them into the center of the open field, the top zone of a novel tank diving arena, or the white compartment of a light/dark test, simply because there are no other zones to move in. In the zMCSF, the risky areas only comprise a small part of the arena, therefore a move into this area may be a more active choice for exploration versus shelter seeking. Hence the zMCSF may allow for a clearer separation between locomotory and explorative activity. Indeed, a study on acute ethanol exposure in male Wistar rats using the MCSF arena showed motor stimulative effects at a low ethanol dose (0.5 g/kg i. p.) and sedative effects at dose (1.5 g/kg i. p.) not commonly reported to induce sedation in conventional behavioral tests (Karlsson and Roman, 2016).

### 4.3 On the Use of the AB Strain

Our results stand in contrast to a study by Souza *et al.* (2021), where a different strain of zebrafish (short-fin zebrafish from a local pet store in Brazil) was exposed to 0, 0.25, 0.5 or 1.0% (v/v) ethanol for one hour, and subsequently tested in a novel object test (Souza et al., 2021). Both sexes showed a reduction in locomotory activity in response to the 1.0% dose. While control females spent less time in the center of the arena than control males, 0.25% exposed females spent equal time in the center as males. Compared to males exposed to the same dose, 0.5% exposed females swam faster towards and kept a greater distance to, the novel object and spent more time in the peripheral zone. The results were interpreted as an anxiolytic effect of 0.5% ethanol in females, that was absent in males (Souza et al., 2021). One explanation for the discrepancy between the studies may lie in the use of different strains. It has been noted before that AB zebrafish in general show aberrant behavior (Gerlai et al., 2008; Gorissen et al., 2015; Vossen et al., 2020) and more specifically, display a distinct response to ethanol in comparison to other strains, both in terms of behavior and monoamine neurotransmitter release (Gerlai et al., 2008; Gerlai et al., 2009). Nonetheless, most mutant strains are derived from the AB strain, which makes the characterization of the pharmacological responses in this specific strain important. Furthermore, it seems that results obtained on AB zebrafish are coherent within this strain. For example, similar sex-specific effects as found herein were detected in AB zebrafish exposed to environmental concentrations (μg L^−1^ range) of the anxiolytic benzodiazepine oxazepam (Vossen et al., 2020). AB males showed a dose-dependent increase in duration in the bottom of a diving arena and an associated decrease in velocity, indicative of a sedative effect, while AB females were largely unaffected at all concentrations (Vossen et al., 2020).

### 4.4 Conclusions

We found significant sex differences in the effect of acute ethanol exposure (1 or 2% (v/v) for 30 min) on adult zebrafish behavior in a complex test arena, the zMCSF. Females were more tolerant, showing mixed depressive/stimulatory effects on locomotory behavior for the 2% dose. Males displayed clear signs of sedation as indicated by reduced activity and exploration and retreat to the sheltered area under the 2% dose, and to a lesser extend under 1% exposure. Our findings emphasize the importance of explicitly including effects of sex (Souza et al., 2021) and time course (Tran and Gerlai, 2013; Pannia et al., 2014) in analyses of behavioral experiments on adult zebrafish. The use of the zMCSF test may have enabled a clearer distinction between locomotory activity and risk-taking/shelter seeking behavior. However, further pharmacological validation of the zMCSF test is needed.

## Data Availability

The data generated for this study can be found on figshare with the identifier https://doi.org/10.6084/m9.figshare.18258962.

## Abbreviations

CENT, Center zone of the zMCSF; CIRC, Central circle zone of the zMCSF; CORN, Corner zone of the zMCSF; CORR1, Corridor 1 zone of the zMCSF; CORR2, Corridor 2 zone of the zMCSF; CORRS, Corridor 1, corner and corridor 2 of the zMCSF; DCR, Dark corner roof zone of the zMCSF; MCSF, Multivariate Concentric Square Field™; NTDT, Novel Tank Diving Test; PCA, Principal Component Analysis; RAMP, Inclined ramp of the zMCSF; REST, The part of the zMCSF not designated to any other zone; START, Start zone of the zMCSF; zMCSF, Zebrafish Multivariate Concentric Square Field™.
