# Supplementary material for: Sex-Specific Effects of Acute Ethanol Exposure on Locomotory Activity and Exploratory Behavior in Adult Zebrafish (Danio rerio)
Source: Front Pharmacol. 2022 Jun 2;13:853936. doi: 10.3389/fphar.2022.853936 (PMC9201571; doi:10.3389/fphar.2022.853936)
Supplement: Supplementary file 1 [file DataSheet2.PDF]

Supplementary Material

1 Supplementary Figures

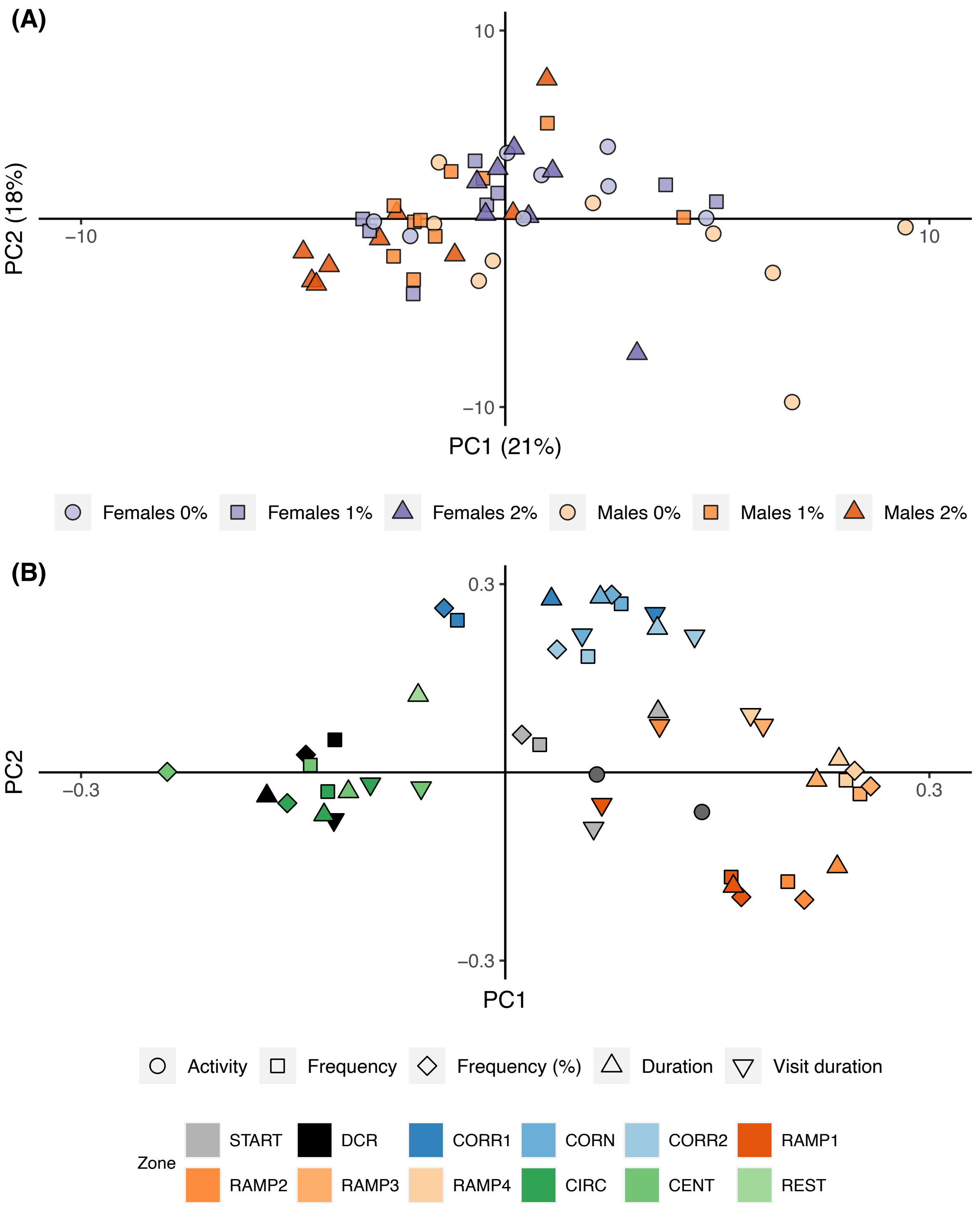

Supplementary Figure 1. Visualized results from the Principal Component Analysis (PCA) on the behavior of male and female AB zebrafish in the zMCSF. (A) score plot and **(B)** loading plot. Colors in (A) indicate the Dose/Sex group: females (purple colors) and males (orange colors) exposed to 0% (controls; light shades), 1% ethanol (medium dark shades) or 2% ethanol (dark shades). Colors in (B) correspond to the zones of the zMCSF as indicated in Figure 1B of the manuscript main text. Abbreviations: CENT, center; CIRC, central circle; CORN, corner; CORR, corridor; DCR, dark corner roof; REST, the part of the arena not designated to any other zone.

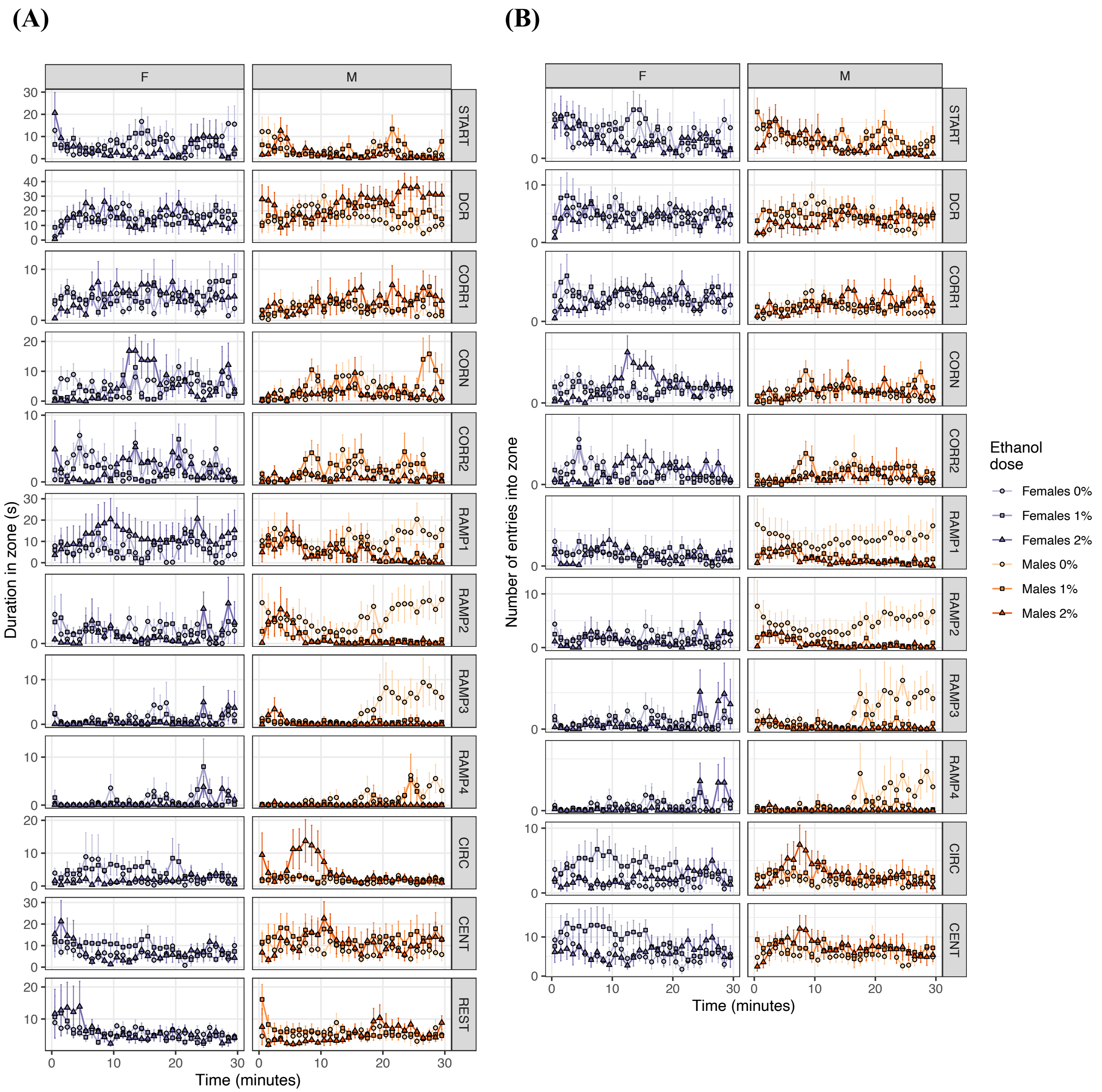

Supplementary Figure 2. Exploration of the zMCSF arena over time. (A) Duration in zone (in seconds) over time (in minutes) and **(B)** frequency of zone entries over time (in minutes), faceted by sex and zone of the zMCSF. Points and errorbars represent means ± SEM per Dose/Sex group. Colors represent sex (female, F, in purple colors and male, M, in orange colors). Color shades represent ethanol dose; light shades 0%, medium dark shades 1% and dark shades 2%. Zone abbreviations: CENT, center; CIRC, central circle; CORN, corner; CORR, corridor; DCR, dark corner roof; REST, the part of the arena not designated to any other zone.

2 Supplementary Tables

Supplementary Table 1. Descriptive statistics for the behavioral variables summarized over the whole 30-minute zMCSF test, per Dose/Sex group. Sample size: the number of fish included in this Dose/Sex group. Total activity: the sum of all frequencies (total number of zone entries). Zones explored: the number of zones entered. Fully explored: the number of animals that had been in all 11 zones of the arena (i.e. excluding the REST zone). All numbers indicate mean ± SEM, except for latency where numbers present median (25% quartile – 75% quartile), the more accurate summarizing statistic for this non-normally distributed variable.

		**Females**			**Males**		
**Zone**	**Sex**	**0%**	**1%**	**2%**	**0%**	**1%**	**2%**
**WHOLE**	**Sample size**	8	8	7	9	10	9
**ARENA**	**Distance moved (cm)**	9494 ± 859	11008 ± 731	9435 ± 800	11118 ± 1421	9296 ± 942	7495 ± 607*
	**Velocity (cm s^{-1})**	5.3 ± 0.5	6.1 ± 0.4	5.3 ± 0.4	6.2 ± 0.8	5.2 ± 0.5	4.2 ± 0.3*
	**Total activity**	712 ± 56	916 ± 107	705 ± 126	843 ± 78	726 ± 106	610 ± 68
	**Zones explored**	11.0 ± 0.0	10.5 ± 0.1	10.3 ± 0.2	10.2 ± 0.2	10.6 ± 0.2	8.3 ± 0.4
	**Fully explored**	8	5	5	6	9	3
**START**	**Duration (s)**	212 ± 57	178 ± 57	133 ± 48	104 ± 34	112 ± 32	68 ± 17
	**Duration per visit (s)**	2 ± 0	2 ± 0	6 ± 5	2 ± 1	1 ± 0	1 ± 0
	**Frequency**	93 ± 27	106 ± 26	68 ± 24	56 ± 12	89 ± 20	54 ± 14
	**Frequency (%)**	13 ± 3	12 ± 3	9 ± 3	7 ± 1	12 ± 1	9 ± 2
	**Latency (s)**	3 (0 - 14)	11 (0 - 117)	0 (0 - 2)	13 (2 - 146)	19 (11 - 40)	55 (0 - 95)
**DCR**	**Duration (s)**	502 ± 163	431 ± 166	433 ± 124	427 ± 116	563 ± 143	748 ± 174
	**Duration per visit (s)**	3 ± 0	3 ± 1	3 ± 1	3 ± 1	4 ± 1	13 ± 6**[‡‡,$$$]
	**Frequency**	138 ± 33	141 ± 44	121 ± 27	114 ± 24	141 ± 30	102 ± 25
	**Frequency (%)**	20 ± 5	16 ± 5	16 ± 3	15 ± 4	20 ± 5	19 ± 6
	**Latency (s)**	48 (17 - 107)	4 (2 - 40)	69 (14 - 202)	102 (6 - 259)	2 (1 - 8)	6 (3 - 51)
**CORR1**	**Duration (s)**	119 ± 17	146 ± 33	137 ± 30	62 ± 13	88 ± 21	110 ± 58
	**Duration per visit (s)**	2 ± 0	1 ± 0	1 ± 0	1 ± 0	1 ± 0	1 ± 0
	**Frequency**	82 ± 10	102 ± 22	90 ± 18	54 ± 13	65 ± 14	71 ± 27
	**Frequency (%)**	12 ± 1	12 ± 3	12 ± 2	7 ± 2	8 ± 2	11 ± 3
	**Latency (s)**	46 (9 - 94)	25 (3 - 57)	75 (41 - 217)	263 (150 - 329)	5 (2 - 34)	100 (60 - 223)

**CORN**	**Duration (s)**	163 ± 51	123 ± 35	158 ± 49	93 ± 42	125 ± 39	79 ± 52
	**Duration per visit (s)**	4 ± 1	3 ± 1	3 ± 1	3 ± 1	3 ± 1	2 ± 1
	**Frequency**	35 ± 5	34 ± 8	45 ± 10	22 ± 5	35 ± 8	29 ± 20
	**Frequency (%)**	5 ± 1	4 ± 1	6 ± 2	3 ± 1	5 ± 1	4 ± 2
	**Latency (s)**	100 (79 - 171)	57 (29 - 298)	613 (137 - 672)	568 (223 - 945)	29 (6 - 355)	1375 (241 - 1800) ‡
**CORR2**	**Duration (s)**	74 ± 19	42 ± 13	55 ± 14	35 ± 11	60 ± 21	17 ± 7^{‡,S}
	**Duration per visit (s)**	4 ± 1	2 ± 0	3 ± 1	2 ± 1	2 ± 0	1 ± 0
	**Frequency**	19 ± 4	15 ± 4	22 ± 8	14 ± 5	22 ± 5	12 ± 4
	**Frequency (%)**	3 ± 1	2 ± 0	3 ± 1	2 ± 1	3 ± 1	2 ± 1
	**Latency (s)**	130 (86 - 188)	218 (51 - 758)	611 (117 - 670)	733 (305 - 944)	178 (8 - 421)	914 (243 - 1800)
**RAMP1**	**Duration (s)**	183 ± 53	222 ± 49	368 ± 225	368 ± 132	145 ± 35	138 ± 54*
	**Duration per visit (s)**	5 ± 1	5 ± 1	8 ± 4	4 ± 0	5 ± 1	4 ± 1
	**Frequency**	41 ± 12	47 ± 9	38 ± 8	122 ± 62	31 ± 7*	23 ± 9***
	**Frequency (%)**	5 ± 1	5 ± 1	10 ± 6	12 ± 5	4 ± 1	3 ± 1**
	**Latency (s)**	80 (8 - 176)	103 (29 - 264)	206 (46 - 416)	149 (5 - 737)	135 (10 - 283)	245 (64 - 631)
**RAMP2**	**Duration (s)**	45 ± 17	52 ± 13	35 ± 7	123 ± 39	24 ± 9	22 ± 13**
	**Duration per visit (s)**	1 ± 0	1 ± 0	1 ± 0	1 ± 0	1 ± 0	1 ± 0
	**Frequency**	45 ± 15	47 ± 12	39 ± 7	147 ± 61	26 ± 9***	18 ± 10***
	**Frequency (%)**	6 ± 2	5 ± 1	10 ± 6	15 ± 5	4 ± 1**	2 ± 1***^{,SS}
	**Latency (s)**	84 (9 - 223)	104 (31 - 332)	312 (51 - 593)	208 (9 - 746)	134 (18 - 292)	480 (73 - 1800)
**RAMP3**	**Duration (s)**	36 ± 16	26 ± 10	19 ± 8	103 ± 54	11 ± 7	9 ± 6**
	**Duration per visit (s)**	1 ± 0	1 ± 0	1 ± 0	1 ± 0	1 ± 0	1 ± 0
	**Frequency**	30 ± 8	26 ± 10	24 ± 8	78 ± 27	15 ± 7**	6 ± 3***^{,S}
	**Frequency (%)**	4 ± 1	3 ± 1	3 ± 1	9 ± 3	2 ± 1**	1 ± 0***
	**Latency (s)**	251 (89 - 542)	173 (31 - 499)	454 (198 - 603)	526 (9 - 747)	255 (47 - 729)	1800 (102 - 1800)
**RAMP4**	**Duration (s)**	24 ± 10	24 ± 15	11 ± 4	46 ± 19	14 ± 11	1 ± 1*
	**Duration per visit (s)**	1 ± 0	1 ± 0	1 ± 0	1 ± 0	1 ± 0	0 ± 0
	**Frequency**	14 ± 4	10 ± 6	10 ± 4	36 ± 15	6 ± 3**	1 ± 1***^{,SS}
	**Frequency (%)**	2 ± 1	1 ± 1	1 ± 1	4 ± 2	1 ± 0*	0 ± 0**
	**Latency (s)**	421 (220 - 796)	818 (344 - 1576)	676 (562 - 1722)	751 (47 - 998)	753 (202 - 1243)	1800 (1249 - 1800)
**CIRC**	**Duration (s)**	75 ± 27	132 ± 55	54 ± 17	45 ± 14	68 ± 12	128 ± 38
	**Duration per visit (s)**	2 ± 1	1 ± 0	1 ± 0	1 ± 0	1 ± 0	2 ± 1

	**Frequency**	52 ± 7	118 ± 35	66 ± 19	53 ± 14	83 ± 14	85 ± 20
	**Frequency (%)**	7 ± 1	12 ± 3	8 ± 2	7 ± 2	12 ± 3	14 ± 3
	**Latency (s)**	11 (2 - 24)	34 (13 - 117)	97 (4 - 325)	307 (4 - 453)	36 (15 - 77)	57 (12 - 176)
**CENT**	**Duration (s)**	183 ± 27	270 ± 68	214 ± 46	240 ± 109	407 ± 140	328 ± 60
	**Duration per visit (s)**	1 ± 0	1 ± 0	1 ± 0	1 ± 1	3 ± 1	2 ± 0
	**Frequency**	165 ± 19	269 ± 63	181 ± 47	147 ± 25	212 ± 37	210 ± 30
	**Frequency (%)**	23 ± 2	27 ± 4	22 ± 4	19 ± 4	29 ± 3	35 ± 3
	**Latency (s)**	0 (0 - 1)	8 (0 - 27)	2 (0 - 29)	1 (0 - 125)	3 (0 - 17)	56 (3 - 166)
**REST**	**Duration (s)**	184 ± 22	156 ± 26	180 ± 51	152 ± 33	183 ± 34	152 ± 15

* Different from control group of the same sex with P<0.05
** Different from control group of the same sex with P<0.01
*** Different from control group of the same sex with P<0.001
‡ Different from low dose group (1%) of the same sex with P<0.05
‡‡ Different from low dose group (1%) of the same sex with P<0.01
‡‡‡ Different from low dose group (1%) of the same sex with P<0.001
^{S} Different from females of the same dose with P<0.05
^{SS} Different from females of the same dose with P<0.01
^{SSS} Different from females of the same dose with P<0.001

Supplementary Table 2. Results from the models on the data from the whole 30-minute zMCSF test. Models of locomotory activity (distance moved, velocity and total activity) contained fixed effects of Dose, Sex and their interaction. Models of explorative behavior (duration, duration per visit, frequency, frequency (%) and latency) additionally contained a fixed effect of Zone, plus all interactions between fixed effects.

Response	Explanatory	Test statistic	df	p-value	
Distance moved (cm)	Dose	F=2.483	2, 45	0.095	.
	Sex	F=0.714	1, 45	0.403	
	Dose × Sex	F=2.151	2, 45	0.128	
Velocity (cm s^{-1})	Dose	F=2.481	2, 45	0.095	.
	Sex	F=0.71	1, 45	0.404	
	Dose × Sex	F=2.171	2, 45	0.126	
Total activity	Dose	χ^2=3.234	2, 48	0.199	
	Sex	χ^2=0.477	1, 47	0.490	
	Dose × Sex	χ^2=2.781	2, 45	0.249	
Number of zones explored	Dose	χ^2=2.113	2, 48	0.357	
	Sex	χ^2=0.866	1, 47	0.352	
	Dose × Sex	χ^2=1.002	2, 45	0.606	
Fully explored	Dose	χ^2=4.715	2, 48	0.095	.
	Sex	χ^2=1.225	1, 47	0.268	
	Dose × Sex	χ^2=7.492	2, 48	0.024	*
Duration in zone (s)	Zone	F=45.204	11, 495	<0.001	***
	Dose	F=1.664	2, 45	0.201	
	Sex	F=3.617	1, 45	0.064	.
	Zone × Dose	F=1.228	22, 495	0.218	
	Zone × Sex	F=1.593	11, 495	0.097	.
	Dose × Sex	F=0.417	2, 45	0.661	
	Zone × Dose × Sex	F=1.96	22, 495	0.006	**
Duration per visit (s)	Zone	F=46.052	10, 410	<0.001	***
	Dose	F=0.469	2, 46	0.628	
	Sex	F=0.393	1, 46	0.534	
	Zone × Dose	F=0.688	20, 410	0.839	
	Zone × Sex	F=2.689	10, 410	0.003	**
	Dose × Sex	F=0.226	2, 46	0.799	
	Zone × Dose × Sex	F=1.226	20, 410	0.228	
Frequency in zone	Zone	χ^2=480.587	10, 493	<0.001	***
	Dose	χ^2=7.169	2, 45	0.028	*
	Sex	χ^2=2.295	1, 45	0.130	
	Zone × Dose	χ^2=58.418	20, 493	<0.001	***
	Zone × Sex	χ^2=4.892	10, 493	0.898	
	Dose × Sex	χ^2=4.950	2, 45	0.084	.
	Zone × Dose × Sex	χ^2=55.734	20, 493	<0.001	***
Frequency in zone (%)	Zone	χ^2=494.668	10, 493	<0.001	***

	Dose	χ^2=2.780	2, 45	0.247	
	Sex	χ^2=1.615	1, 45	0.204	
	Zone × Dose	χ^2=51.980	20, 493	<0.001	***
	Zone × Sex	χ^2=6.213	10, 493	0.797	
	Dose × Sex	χ^2=3.821	2, 45	0.148	
	Zone × Dose × Sex	χ^2=59.868	20, 493	<0.001	***
Latency to zone (s)	Zone	F=31.573	10, 450	<0.001	***
	Dose	F=4.817	2, 45	0.013	*
	Sex	F=4.333	1, 45	0.043	*
	Zone × Dose	F=1.325	20, 450	0.158	
	Zone × Sex	F=0.915	10, 450	0.519	
	Dose × Sex	F=2.098	2, 45	0.135	
	Zone × Dose × Sex	F=0.725	20, 450	0.801	
